# Socioeconomic variation in the prevalence of pain by anatomical sites among middle-aged and older adults in India: a cross-sectional study

**DOI:** 10.1186/s12877-024-04780-1

**Published:** 2024-02-27

**Authors:** Amit Kumar Goyal, Sanjay K. Mohanty

**Affiliations:** International Institute for Population Sciences, Mumbai, India

**Keywords:** Pain, Back pain, Joint pain, Public health, Quality of life

## Abstract

**Background:**

Pain is a significant global public health concern, particularly among individuals aged 45 and above. Its impact on the overall lifestyle of the individuals varies depending on the affected anatomical parts. Despite its widespread impact, there is limited awareness of the attributes of pain, making effective pain management challenging, particularly in India. This study aims to estimate the prevalence and variation in pain in different anatomical sites among middle-aged and older adults in India.

**Methods:**

A cross-sectional design was employed, utilising data from the first wave of the Longitudinal Aging Study in India (LASI), 2017–2018. The age-sex adjusted prevalence of pain by anatomical sites (the back, joints, and ankles) was estimated using a multivariate logistic regression model.

**Results:**

47% of individuals aged 45 years and above reported joint pain, 31% reported back pain and 20% suffered from ankle or foot pain. The prevalence of pain at all the anatomical sites increased with age and was reported higher among females. Relative to respondents aged 45–59 years, those aged 75 years and older exhibited a 41% higher likelihood of experiencing back pain (AOR: 1.41, 95% CI: 1.19–1.67), a 67% higher likelihood of joint pain (AOR: 1.67, 95% CI: 1.49–1.89), and a 32% higher likelihood of ankle/foot pain (AOR: 1.32, 95% CI: 1.16–1.50). In comparison to males, females had a 56% higher likelihood of encountering back pain (AOR: 1.56, 95% CI: 1.40–1.74), a 38% higher likelihood of joint pain (AOR: 1.38, 95% CI: 1.27–1.50), and a 35% higher likelihood of ankle/foot pain (AOR: 1.35, 95% CI: 1.17–1.57). We also found significant regional variations in pain prevalence, with higher rates in the mountainous regions of India.

**Conclusion:**

This research highlights the high burden of pain in major anatomical sites among middle-aged and older adults in India and emphasises the need for increased awareness and effective pain management strategies.

**Supplementary Information:**

The online version contains supplementary material available at 10.1186/s12877-024-04780-1.

## Introduction

Pain among middle-aged and older populations aged 45 years and above is a significant global public health concern, impacting their daily activities and functional abilities. Persistent pain becomes chronic and adversely affects the quality of life [1–3]. Despite the significant impact of pain, there is limited information on pain prevalence by anatomical sites in developing countries, making pain management challenging. Major anatomical sites include the back, the joints, and the ankles. Back pain can lead to functional limitations and disability among older adults [4], limiting their ability to work [5], participate in social activities, and carry out basic self-care activities [6]. Joint pain also limits their mobility and makes it challenging for them to perform physical activities such as walking, climbing stairs, and exercising. It can also lead to a sedentary lifestyle and contribute to other health issues such as obesity, cardiovascular disease, and depression. On its part, ankle/foot pain impairs mobility and balance and increases the risk of falls [7], making it difficult for older adults to maintain independence and participate in social and economic activities.

Pain is a complex phenomenon and is influenced by various factors, including death or damage of tissue, injury, inflammation, nerve damage, stress, depression, ageing, obesity, lifestyle and behavioural factors. Social interactions and personal characteristics can also shape an individual’s perception of pain, leading to a diverse range of experiences and expressions of pain. Literature suggests significant heterogeneity in pain prevalence based on various sociodemographic characteristics [8–13]. Therefore, it is essential to consider these socioeconomic determinants of health when assessing pain prevalence by anatomical sites and developing interventions to address it effectively.

India is undergoing a rapid demographic transition, marked by significant shifts in the age and sex composition that span geographical, economic, and social boundaries. Concurrently, the nation is experiencing an epidemiological transition characterised by increased life expectancy and an overall trend towards a longer lifespan. Despite these significant changes, comprehensive national-level data on pain has only recently become available. The existing studies on pain are based on small-scale, non-representative samples that need to adequately reflect the national landscape [14]. There is an absence of nationally representative studies explicitly investigating the prevalence of pain at major anatomical sites. Using data from a nationally representative survey, this study aims to estimate the prevalence of pain in different anatomical sites among middle-aged and older adults in India based on sociodemographic characteristics.

## Methods

### Data

The present study used data from the first wave of the Longitudinal Aging Study in India (LASI), conducted in 2017–2018. LASI is a nationally representative, prospective cohort study that surveyed adults aged 45 years and above and their spouses, regardless of age. It covered a representative sample of 42,949 households and 72,250 individuals across 36 states and union territories in India. It used stratified, multistage cluster sampling to select non-institutional households. The detailed sampling design can be found elsewhere [15]. Face-to-face interviews were conducted with all individuals aged 45 years and above and their spouses residing in the selected households. Our analytical sample includes 58,502 individuals aged 45 years and above. The LASI survey posed a series of questions to the respondents, delving into their experiences with pain. The inquiry encompassed several aspects, such as whether they experienced pain often, how frequently the pain occurred, whether they took any medication or therapy for pain alleviation, and in what locations they had experienced persistent or troublesome pain during the two years before the survey date.

### Outcome variables

The analysis used three binary pain variables: back pain, joint pain, and ankle/foot pain. These variables were derived from self-reported data collected using a list of persistent or troublesome conditions. The respondents were asked to indicate whether they had experienced any of the following conditions during the two years before the survey: back pain or problem (back pain), pain or stiffness in joints (joint pain), and persistent swelling in feet or ankles (ankle pain).

### Covariates

We estimated variations in pain prevalence across a range of socioeconomic characteristics, including age (‘45–59’ years, ’60–74’ years, ‘75+’ years), sex (‘male’, ‘female’), place of residence (‘rural’, ‘urban’), years of education (‘no education’, ‘<5 years’, ‘5–9 years’, ‘≥ 10 years’), married (‘no’, ‘yes’), caste (‘SC’, ‘ST’, ‘OBC’, ‘others’); religion (‘Hindu’, ‘Muslim’, ‘Christian’, ‘others’), monthly per capita income (MPCE) quintiles (‘poorest’, ‘poorer’, ‘middle’, ‘richer’, ‘richest’), currently working (‘no’, ‘yes’), BMI level (‘underweight’, ‘normal’, ‘overweight’, ‘obese’), and health behaviours such as history of smoking (‘no’, ‘yes’), alcohol consumption (‘no’, ‘yes’) and physically active (‘no’, ‘yes’). By examining these factors, we sought to identify patterns of and variation in pain prevalence among different population subgroups.

### Statistical analysis

We accounted for the survey design parameters to ensure robust estimation, including the stratified two-stage cluster design, the sampling weights, and the survey strata by applying *surveyset*. We adjusted the estimate of pain prevalence for age and sex to improve the accuracy of our results (see the supplementary text 1 for details). To estimate the prevalence of pain across various anatomical sites, we employed a multivariate logistic regression model and reported the results as percentages with a 95% confidence interval. We also took steps to enhance the reliability of the models and assess their generalizability. Appendices 1 and 2 show subsets of data specific to ages 45–59 years and 60 + years, respectively.

## Results

### Sample characteristics

Table 1 shows the socio-demographic characteristics of the study population. Out of 27,851 people with joint pain, the majority were 45–59 years old (46.4%), females (60.1%), resided in rural areas (67.9%), had no schooling (52.9%), were currently married (72.7%), belonged to the other backward caste (37.5%), and were Hindu (71.6%). The sample composition remained similar for the other two anatomical sites: back pain and ankle/foot pain.

**Table 1:** Socio-demographic characteristics of middle aged and older adults with and without pain (*N* = 59,502), India, 2017-18

Socio-Demographic Characteristics	Any Pain (*N* = 35,976) N (%)	Back Pain (*N* = 20,721) N (%)	Joint Pain (*N* = 27,851) N (%)	Ankle/Foot Pain (*N* = 10,193) N (%)
**Age**				
45–59	17,408 (48.4)	10,139 (48.9)	12,912 (46.4)	4690 (46.0)
60–74	14,556 (40.5)	8193 (39.5)	11,637 (41.8)	4255 (41.7)
75 and above	4012 (11.2)	2389 (11.5)	3302 (11.9)	1248 (12.2)
**Sex**				
Male	14,722 (40.9)	8117 (39.2)	11,028 (39.6)	3698 (36.3)
Female	21,254 (59.1)	12,604 (60.8)	16,823 (60.4)	6495 (63.7)
**Residence**				
Rural	24,294 (67.5)	14,398 (69.5)	18,910 (67.9)	6849 (67.2)
Urban	11,682 (32.5)	6323 (30.5)	8941 (32.1)	3344 (32.8)
**Years of Education**				
No schooling	18,393 (51.1)	10,898 (52.6)	14,736 (52.9)	5419 (53.2)
< 5 years	4288 (11.9)	2470 (11.9)	3281 (11.8)	1158 (11.4)
5–9 years	7838 (21.8)	4415 (21.3)	5878 (21.1)	2173 (21.3)
≥ 10 years	5457 (15.2)	2938 (14.2)	3956 (14.2)	1443 (14.2)
**Currently Married**				
No	9808 (27.3)	5692 (27.5)	7906 (28.4)	2952 (29.0)
Yes	26,168 (72.7)	15,029 (72.5)	19,945 (71.6)	7241 (71.0)
**Caste**				
Scheduled caste	5910 (16.4)	3215 (15.5)	4688 (16.8)	1788 (17.5)
Scheduled tribe	6680 (18.6)	4794 (23.1)	4836 (17.4)	1259 (12.4)
Other Backward Class	13,491 (37.5)	7302 (35.2)	10,688 (38.4)	3956 (38.8)
Others	9895 (27.5)	5410 (26.1)	7639 (27.4)	3190 (31.3)
**Religion**				
Hindu	25,691 (71.4)	13,953 (67.3)	20,222 (72.6)	76.85 (76.9)
Muslim	4266 (11.9)	2533 (12.2)	3362 (12.1)	1193 (11.7)
Christian	4089 (11.4)	3092 (14.9)	2831 (10.2)	600 (5.9)
Others	1930 (5.4)	1143 (5.5)	1436 (5.2)	567 (5.6)
**MPCE quintile**				
Poorest	6930 (19.3)	3921 (18.9)	5391 (19.4)	1843 (18.1)
Poorer	7259 (20.2)	4143 (20.0)	5621 (20.2)	2012 (19.7)
Middle	7178 (20.0)	4050 (19.6)	5574 (20.0)	2019 (19.8)
Richer	7342 (20.4)	4234 (20.4)	5619 (20.2)	2160 (21.2)
Richest	7267 (20.2)	4373 (21.1)	5646 (20.3)	2159 (21.2)
**Currently Working**				
No	20,769 (57.7)	11,790 (56.9)	16,533 (59.4)	6543 (64.2)
Yes	15,207 (42.3)	8931 (43.1)	11,318 (40.6)	3650 (35.8)
**BMI**				
Underweight (≤ 18.5)	6379 (17.7)	3812 (18.4)	4875 (17.5)	1703 (16.7)
Normal (18.5–25.0)	18,430 (51.2)	10,902 (52.6)	14,092 (50.6)	4757 (46.7)
Overweight (25.0–30.0)	8213 (22.8)	4487 (21.7)	6464 (23.2)	2563 (25.1)
Obese (> 30)	2954 (8.2)	1520 (7.3)	2420 (8.7)	1170 (11.5)
**Smoking history**				
No	22,794 (63.4)	12,906 (62.3)	17,837 (64.0)	6635 (65.1)
Yes	13,182 (36.6)	7815 (37.7)	10,014 (36.0)	3558 (34.9)
**Alcohol History**				
No	29,779 (82.8)	17,079 (82.4)	23,211 (83.3)	8714 (85.5)
Yes	6197 (17.2)	3642 (17.6)	4640 (16.7)	1479 (14.5)
**Physically Active**				
No	14,322 (46.0)	8304 (40.1)	11,010 (39.5)	4362 (42.8)
Yes	21,654 (41.7)	12,417 (59.9)	16,841 (60.5)	5831 (57.2)

note: MPCE is an abbreviation for monthly per capita consumption expenditure and BMI refers to body mass index

### Pain prevalence

**Fig. 1:**
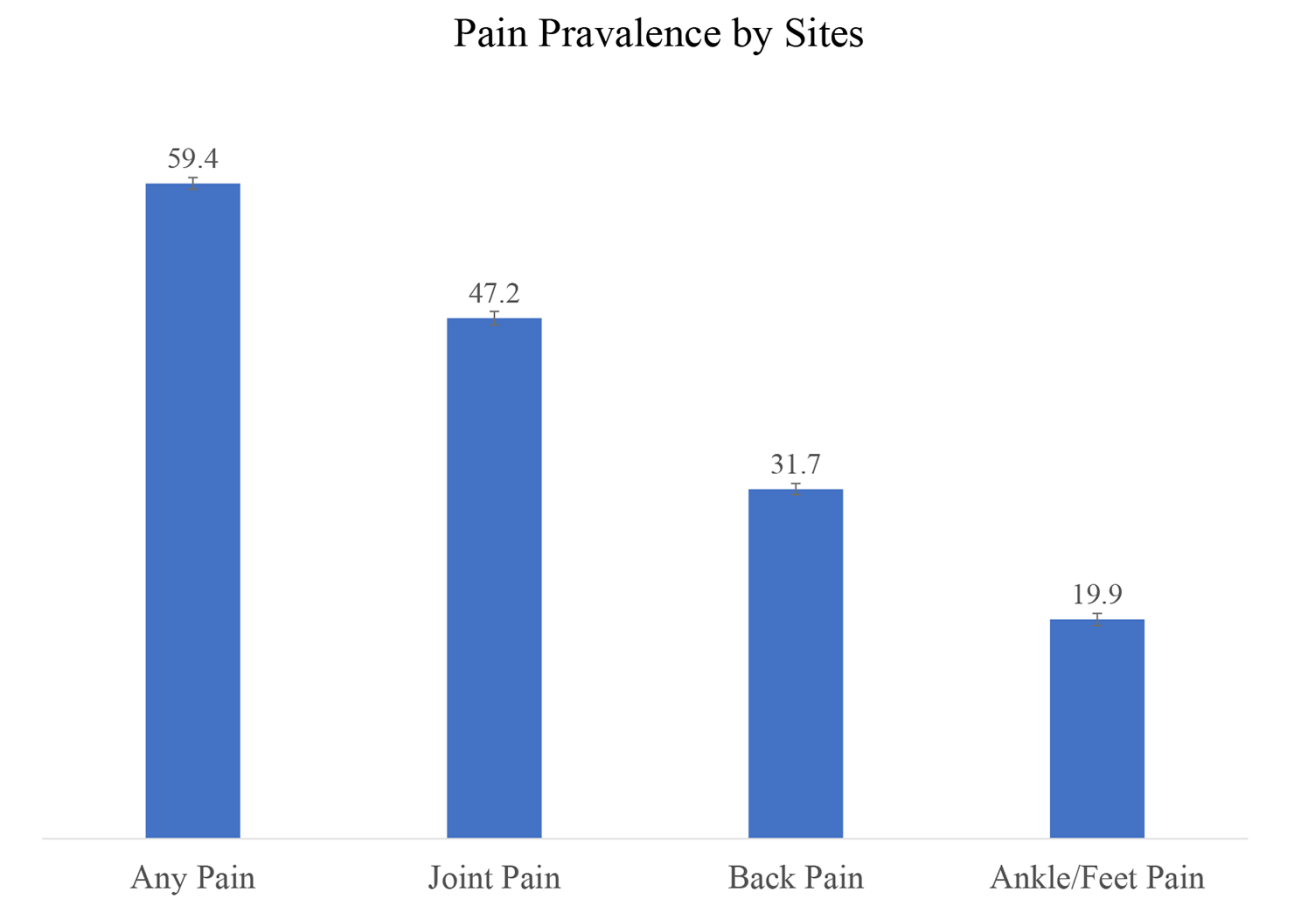
Age-sex adjusted prevalence of pain by sites

Table 2; Fig. 1 show the age-sex adjusted pain prevalence at different anatomical sites among middle-aged and older adults in India. Approximately half of India’s middle-aged and older adults reported joint pain, 31.7% (95% CI: 30.68–32.69) reported back pain, and 19.87% (95% CI: 18.77–20.97) had ankle/foot pain. The prevalence of pain at all the anatomical sites varied significantly by different socio-demographic factors, such as age, sex, education, BMI, and smoking history.

**Table 2:** Age-sex adjusted prevalence of pain at different sites among middle aged and older adults, India, 2017-18

Socio-Demographic Characteristics	Any Pain	Back Pain	Joint Pain	Ankle/Foot Pain
**Overall**	**59.39 (58.35–60.43)**	**31.68 (30.68–32.69)**	**47.18 (45.95–48.40)**	**19.87 (18.77–20.98)**
**Age**				
45–59	54.46 (53.20–55.71)	28.97 (27.67–30.27)	41.92 (40.35–43.49)	17.05 (16.17–17.93)
60–74	63.72 (62.25–65.20)	33.96 (32.62–35.30)	51.50 (50.00–52.99)	22.70 (20.45–24.96)
75 and above	66.49 (64.12–65.20)	36.13 (33.20–39.05)	55.96 (53.64–58.28)	22.84 (20.89–24.79)
**Sex**				
Male	52.48 (50.95–54.01)	26.35 (25.19–27.51)	40.46 (38.93–41.98)	15.38 (14.56–16.20)
Female	65.21 (64.15–66.27)	36.20 (34.82–37.58)	52.86 (51.58–54.14)	23.70 (21.86–25.54)
**Residence**				
Rural	59.82 (58.72–60.92)	32.37 (31.30–33.44)	47.40 (46.28–48.52)	19.26 (18.35–20.18)
Urban	58.39 (55.98–60.81)	30.09 (27.81–32.36)	46.65 (43.56–49.74)	21.29 (18.55–24.02)
**Years of Education**				
No schooling	61.29 (60.00–62.57)	33.84 (32.35–35.33)	49.35 (47.97–50.72)	20.22 (18.85–21.59)
< 5 years	61.48 (59.61–63.36)	32.95 (30.80–35.09)	48.45 (46.51–50.40)	19.95 (18.29–21.62)
5–9 years	57.64 (55.71–59.56)	28.97 (27.00–30.95)	44.79 (42.79–46.80)	19.76 (17.49–22.03)
≥ 10 years	54.84 (51.19–58.49)	27.38 (24.98–29.79)	42.76 (37.94–47.59)	18.78 (14.46–23.10)
**Currently Married**				
No	57.45 (55.89–59.00)	29.80 (28.14–31.46)	45.89 (44.21–47.57)	19.68 (17.67–21.68)
Yes	60.03 (58.80–61.26)	32.42 (31.23–33.60)	47.64 (46.32–48.95)	19.96 (18.97–20.95)
**Caste**				
Scheduled caste	58.56 (56.46–60.67)	31.49 (29.51–33.48)	46.79 (44.73–48.84)	19.90 (17.96–21.85)
Scheduled tribe	61.14 (58.51–63.77)	35.76 (33.09–38.44)	48.57 (45.62–51.52)	18.65 (15.71–21.59)
Other Backward Class	59.80 (58.15–61.45)	31.52 (30.17–32.87)	48.26 (46.28–50.23)	19.32 (17.31–21.34)
Others	58.71 (57.16–60.26)	30.78 (29.26–32.31)	45.13 (43.57–46.70)	21.20 (20.01–22.38)
**Religion**				
Hindu	59.35 (57.98–60.71)	31.34 (30.28–32.41)	47.32 (45.74–48.89)	20.26 (18.86–21.65)
Muslim	59.15 (56.45–61.84)	33.50 (30.43–36.57)	45.94 (43.57–48.31)	18.58 (16.63–20.53)
Christian	58.44 (49.73–67.15)	33.45 (27.29–39.61)	46.52 (39.56–53.49)	11.96 (8.77–15.16)
Others	61.91 (58.24–65.58)	32.59 (29.83–35.35)	48.26 (43.90–52.63)	21.55 (18.60–24.50)
**MPCE quintile**				
Poorest	56.91 (54.61–59.21)	30.39 (28.69–32.09)	44.96 (42.77–47.15)	16.63 (15.33–17.93)
Poorer	59.71 (57.85–61.57)	31.69 (29.96–33.42)	48.02 (46.08–49.96)	19.31 (17.70–20.92)
Middle	58.49 (56.71–60.26)	29.57 (27.99–31.15)	45.84 (44.00–47.68)	20.35 (18.04–22.66)
Richer	59.02 (56.99–61.05)	31.22 (28.69–33.75)	45.78 (44.09–47.47)	21.53 (19.79–23.27)
Richest	63.39 (60.56–66.23)	36.22 (32.93–39.51)	51.91 (48.21–55.61)	22.07 (20.42–23.72)
**Currently Working**				
No	60.69 (59.52–61.86)	31.50 (30.23–32.77)	49.36 (47.91–50.81)	21.63 (20.16–23.10)
Yes	58.00 (56.35–59.65)	31.92 (30.53–33.30)	44.66 (43.04–46.29)	17.56 (16.48–18.64)
**BMI**				
Underweight (≤ 18.5)	54.37 (52.50–56.24)	30.92 (29.37–32.47)	41.74 (40.15–43.34)	16.65 (15.29–18.02)
Normal (18.5–25.0)	58.08 (56.88–59.27)	31.53 (30.32–32.75)	45.73 (44.48–46.98)	18.20 (17.35–19.06)
Overweight (25.0–30.0)	64.44 (62.27–66.61)	31.30 (28.48–34.12)	52.84 (50.13–55.54)	24.08 (21.31–26.85)
Obese (> 30)	70.02 (66.72–73.31)	36.30 (30.41–42.19)	58.40 (54.32–62.48)	30.07 (25.81–34.34)
**Smoking history**				
No	57.95 (56.33–59.56)	30.38 (28.93–31.82)	46.41 (44.56–48.27)	19.73 (18.31–21.14)
Yes	61.68 (60.30–63.06)	34.01 (32.63–35.40)	48.45 (46.99–49.92)	20.14 (18.98–21.30)
**Alcohol History**				
No	58.86 (57.65–60.08)	31.37 (30.26–32.48)	46.74 (45.34–48.13)	19.92 (18.79–21.06)
Yes	62.16 (60.40–63.92)	33.63 (31.64–35.62)	49.67 (47.79–51.55)	19.53 (17.55–21.51)
**Physically Active**				
No	60.29 (59.02–61.56)	32.58 (31.39–33.76)	47.91 (46.57–49.25)	21.09 (19.91–22.28)
Yes	58.87 (57.37–60.37)	31.17 (29.90–32.44)	46.75 (44.95–48.55)	19.16 (17.53–20.79)

note: MPCE is an abbreviation for monthly per capita consumption expenditure and BMI refers to body mass index

Joint pain was more prevalent among those aged 75 years and above (55.96%), females (52.86%), had no schooling (49.35%), belonged to the richest MPCE quintile (51.91%), obese (58.40%), history of smoking (48.45%) and alcohol consumption (49.67%). Likewise, back pain was more prevalent among individuals in the older age groups, with the highest rate among those aged 75 years and above (36.13%), were females (36.20%), had no schooling (33.84%), belonged to the richest MPCE quintile (36.22%), were obese (36.30%) or had a history of smoking (34.01%) and alcohol consumption (33.63%). The prevalence of ankle/foot pain was highest among those aged 75 years and above (22.84%), females (23.70%), had no schooling (20.22%), belonged to the richest MPCE quintile (22.07%), obese (30.07%) and had a history of smoking (20.14%).

### Predictors of pain

Table 3 provides the adjusted odds ratios (AORs), illustrating the association between various socio-demographic factors and the incidence of back pain, joint pain, and ankle/foot pain. When compared to individuals aged 45–59 years, those aged 75 years and older exhibited a 41% higher likelihood of experiencing back pain (AOR: 1.41, 95% CI: 1.19–1.67), joint pain (AOR: 1.67, 95% CI: 1.49–1.89), and ankle/foot pain (AOR: 1.32, 95% CI: 1.16–1.50). In comparison to males, females had a 56% higher likelihood of encountering back pain (AOR: 1.56, 95% CI: 1.40–1.74), a 38% higher likelihood of having joint pain (AOR: 1.38, 95% CI: 1.27–1.50), and 35% higher likelihood of experiencing ankle/foot pain (AOR: 1.35, 95% CI: 1.17–1.57). The AORs for other socio-demographic variables, including residence, years of education, marital status, caste, religion, monthly per capita consumption expenditure (MPCE) quintile, employment status, body mass index (BMI), history of smoking, alcohol consumption, and physical activity, also demonstrated a significant association between these factors and the prevalence of back pain, joint pain, and ankle/foot pain.

**Table 3:** Socio-economic predictors of pain occurrence at different anatomical sites

Socio-Demographic Characteristics	Any Pain	Back Pain	Joint Pain	Ankle/Foot Pain
	AORs (95% CI)	AORs (95% CI)	AORs (95% CI)	AORs (95% CI)
**Age**				
45–59®				
60–74	1.45***(1.35–1.56)	1.26***(1.17–1.37)	1.42***(1.33–1.51)	1.35***(1.20–1.53)
75 and above	1.65***(1.45–1.89)	1.41***(1.19–1.67)	1.67***(1.49–1.89)	1.32***(1.16–1.50)
**Sex**				
Male®				
Female	1.54***(1.41–1.68)	1.56***(1.40–1.74)	1.38***(1.27–1.50)	1.35***(1.17–1.57)
**Residence**				
Rural®				
Urban	0.95(0.85–1.06)	0.99(0.87–1.12)	0.97(0.85–1.10)	1.09(0.95–1.25)
**Years of Education**				
No schooling®				
< 5 years	0.94(0.86–1.02)	0.94(0.83–1.06)	0.89**(0.81–0.98)	0.88*(0.78–1.00)
5–9 years	0.75***(0.67–0.84)	0.76**(0.65–0.89)	0.71***(0.64–0.80)	0.79**(0.67–0.95)
≥ 10 years	0.62***(0.55–0.71)	0.68***(0.58–0.79)	0.61***(0.51–0.71)	0.65**(0.48–0.89)
**Currently Married**				
No®				
Yes	1.08*(1.00–1.16)	1.13**(1.03–1.24)	1.04(0.97–1.12)	0.96(0.85–1.09)
**Caste**				
Scheduled caste®				
Scheduled tribe	1.18*(1.00–1.39)	1.22**(1.05–1.41)	1.15*(0.97–1.35)	1.08(0.86–1.36)
Other Backward Class	1.09*(0.99–1.21)	1.03(0.93–1.14)	1.09*(0.99–1.21)	0.95(0.82–1.10)
Others	1.05(0.92–1.18)	1(0.87–1.15)	0.96(0.85–1.08)	1.05(0.89–1.23)
**Religion**				
Hindu®				
Muslim	0.92(0.81–1.04)	1.07 (0.92–1.24)	0.88**(0.79–0.98)	0.81**(0.68–0.96)
Christian	0.99(0.68–1.44)	1.10 (0.84–1.43)	0.99(0.72–1.35)	0.54**(0.37–0.77)
Others	1.08(0.90–1.29)	1.04 (0.90–1.21)	0.98(0.81–1.20)	0.97(0.79–1.19)
**MPCE quintile**				
Poorest®				
Poorer	1.12**(1.00–1.26)	1.08(0.97–1.20)	1.14**(1.02–1.28)	1.19**(1.03–1.37)
Middle	1.07(0.94–1.22)	1(0.89–1.12)	1.05(0.92–1.19)	1.28**(1.07–1.53)
Richer	1.1(0.96–1.25)	1.09(0.94–1.27)	1.05(0.93–1.18)	1.37**(1.18–1.59)
Richest	1.33**(1.11–1.58)	1.42**(1.16–1.73)	1.35**(1.10–1.64)	1.37**(1.19–1.58)
**Currently Working**				
No®				
Yes	0.86***(0.79–0.93)	0.98(0.91–1.06)	0.79***(0.74–0.85)	0.77***(0.71–0.85)
**BMI**				
Underweight (≤ 18.5) ®				
Normal (18.5–25.0)	1.24***(1.14–1.35)	1.07(0.98–1.17)	1.26***(1.16–1.36)	1.13**(1.03–1.24)
Overweight (25.0–30.0)	1.77***(1.56–2.01)	1.12*(0.98–1.27)	1.82***(1.61–2.04)	1.65***(1.40–1.93)
Obese (> 30)	2.37***(1.97–2.86)	1.45**(1.10–1.90)	2.35***(1.95–2.82)	2.22***(1.83–2.70)
**Smoking history**				
No®				
Yes	1.21***(1.12–1.31)	1.17**(1.06–1.28)	1.13**(1.04–1.22)	1.1**(1.00–1.21)
**Alcohol History**				
No®				
Yes	1.07***(0.98–1.17)	1.02(0.92–1.14)	1.07(0.98–1.17)	0.96(0.84–1.09)
**Physically Active**				
No®				
Yes	0.95 (0.88–1.03)	0.94* (0.87–1.01)	0.98 (0.90–1.07)	0.91 (0.81–1.03)

note: MPCE is an abbreviation for monthly per capita consumption expenditure and BMI refers to body mass index

*** significant at 0.001 level; ** at 0.05 level; * at 0.10 level

### State-level estimates of pain prevalence

**Fig. 2:**
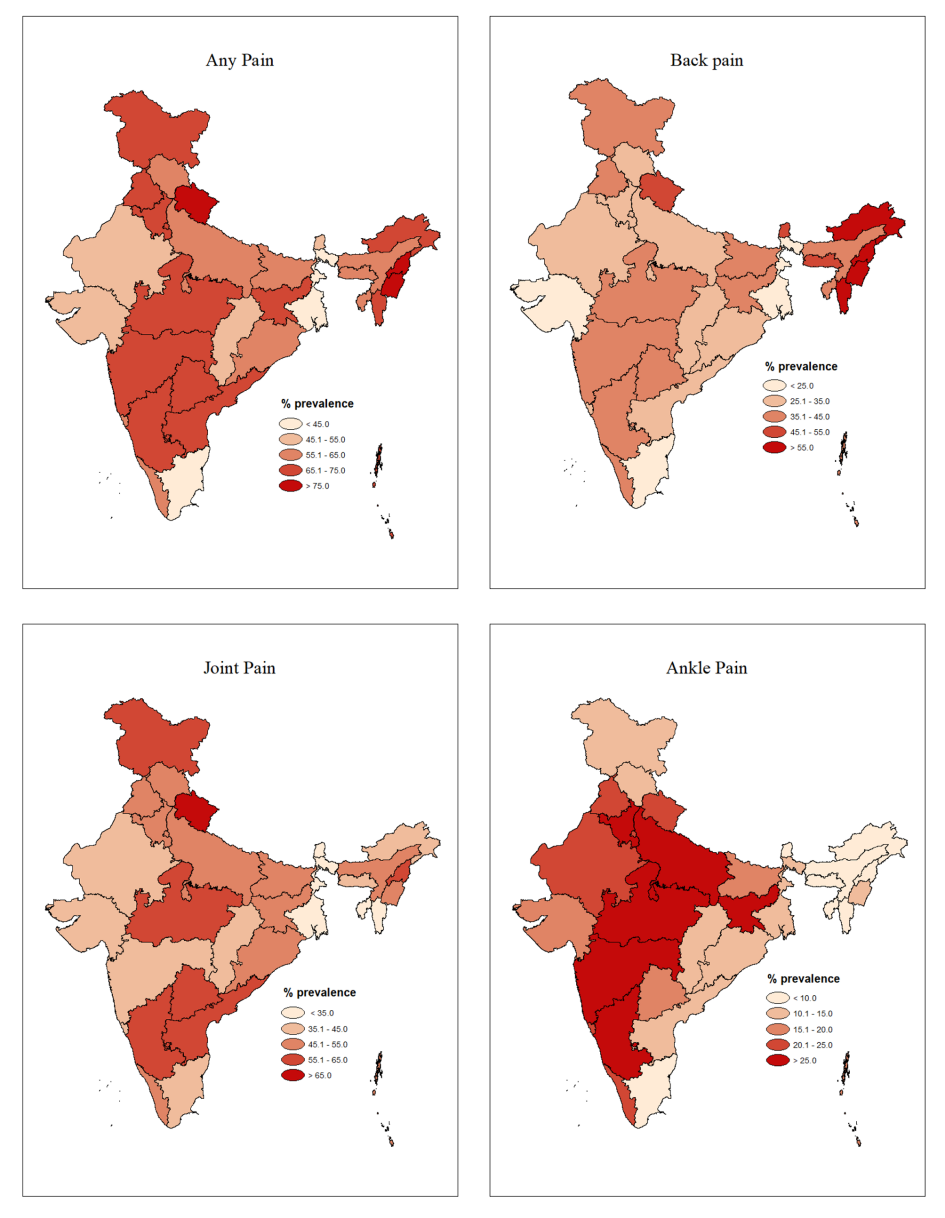
State-level Estimates of chronic pain by sites

Figure 2 shows variations in the prevalence of back pain, joint pain, and ankle/foot pain among different states in India. Joint pain was most prevalent among the older adults of Uttarakhand (67.5%) and least prevalent in West Bengal (26.8%). Back pain was notably more prevalent among the older adults of Manipur (71.2%) than in the rest of the states while being the least prevalent in Tamil Nadu (14.5%). Regarding ankle pain, Madhya Pradesh had the highest prevalence at 32%, while Nagaland reported the lowest incidence at 2.5% (Appendix 3).

## Discussion

This is the first nationally representative study that estimates the prevalence of back pain, joint pain, and ankle pain among middle-aged and older adults in India. It also provides variations in the pain prevalence across sociodemographic characteristics. The salient findings are as follows. First, about half of the individuals aged 45 years and above reported joint pain, one-third reported back pain, and one-fifth suffered from ankle or foot pain. Second, the prevalence of pain at each anatomical site increased consistently with age, was higher in females, reduced with an increase in the years of education, was higher among married individuals, increased with expenditure level, and was higher in case of smoking and physical inactivity. Joint pain and back pain were more prevalent among rural residents, whereas ankle pain was more common among urban residents and those with a history of alcohol consumption. We didn’t find any specific pattern of pain prevalence across caste and religious groups. Third, age, years of education, work status and smoking history were consistent and significant predictors of pain at all the anatomical sites. BMI was significantly associated with only joint pain and ankle/foot pain. Fourth, joint pain, back pain, and ankle/feet pain were most prevalent in Uttarakhand, Manipur, and Madhya Pradesh, respectively, while being least prevalent in West Bengal, Tamil Nadu, and Nagaland, respectively.

The study indicates that pain prevalence across anatomical sites increases with age. Such a high prevalence of pain in older ages can be ascribed to the cumulative effects of wear and tear of tissues in older adults. Nevertheless, it is also crucial to acknowledge that the heterogeneity in pain reporting may be the product of the ageing process [16]. Our study’s estimated prevalence of back pain was lower than the previous estimate of 39% among older adults in low- and middle-income countries [17]. This disparity in estimates may be attributed to differences in the age groups examined; our study focused on those aged 45 years and above, while the prior research was based on individuals aged 50 years and older. Additionally, our study employed a 2-year reference period for pain measurement compared to 30 days [18]. The results of an international consortium of several population-based cohorts also revealed a wide range of prevalence rates for pain, varying from 13 to 36% due to discrepancies in pain definitions [19].

The variations we observed in the pain prevalence estimates across socioeconomic and demographic groups align with findings from existing literature [1, 14, 20]. Consistent with the previous findings, females consistently reported a higher prevalence of pain at all anatomical sites [21]. Postmenopausal women, in particular, often experience more severe disc space narrowing than their male counterparts, resulting in intense back pain [22, 23]. This, coupled with an elevated risk of health conditions contributing to pain and poorer self-rated health, may collectively account for the higher incidence of pain among females [24]. Additionally, females tend to be more willing to acknowledge and report pain [13, 25, 26].

In line with existing research, it was evident that individuals in the obese population were more prone to experiencing joint pain [27]. Obesity is one of the significant risk factors for joint pain and osteoarthritis [28, 29], primarily due to the excessive weight exerting substantial stress on weight-bearing joints, which results in wear and tear and ultimately lead to joint pain [27, 30].

Our estimates of ankle or foot pain were close to an English study on individuals aged 55 years and above [31, 32]. Besides being attributable to demographic factors, ankle/foot pain is attributable to economic conditions as it is more prevalent among individuals with a higher economic status. Such individuals tend to be sedentary, resulting in muscle weakness and reduced flexibility [33]. These factors collectively intensify the strain on the feet and the ankles, leading to pain and discomfort. Furthermore, individuals in the highest wealth quintile may have underlying health conditions contributing to foot and ankle pain, such as arthritis [34], diabetes [35], or circulatory problems.

In examining the variation in pain prevalence at the sub-national level, we found that varying pain reporting extended beyond the national borders, as observed across European countries [36]. India, with its rich tapestry of socio-cultural diversity and economic disparities, reflects a similar pattern within its boundaries [8]. Consistent with the previous findings, our study found the prevalence of joint pain notably elevated in mountainous states like Uttarakhand, Nagaland, and Arunachal Pradesh. This may be attributed to the challenging topography of these regions, which imposes mechanical stress on joints, especially among the elderly population [37]. Beyond the physical factors, numerous socioeconomic elements contribute to this variation in reporting pain across states. First, the language spoken in a particular state and the unique cultural connotations are associated with pain reporting [38]. Secondly, the nature of people’s occupations and their working postures can significantly impact the occurrence of pain [39–41]. Thirdly, lower levels of education in a specific state may perceive higher pain prevalence [42].

The existing healthcare in India doesn’t include pain in its ambit of diseases. Given the chronic nature of pain, we suggest integrating pain treatment and management under the non-communicable disease programs. Our research findings are particularly worrisome as they reveal that pain is already widespread and potentially debilitating, with a higher impact on women, individuals from lower socioeconomic backgrounds, and older adults than their counterparts. Our study can potentially guide national and state governments in shaping health policies and pain management strategies. By shedding light on the extensive prevalence of pain and its social disparities, we aim to emphasise the urgency of recognising pain as one of the major chronic conditions.

This study has significant strengths, including robust statistical estimation methods and using nationally representative data encompassing health and socioeconomic indicators for adults aged 45 years and above. Nevertheless, it is essential to acknowledge certain limitations. First, the study employs a cross-sectional design, which restricts our ability to draw causal inferences. Second, the data source restricts our capacity to comprehensively analyse preventive and palliative care and delve into any underlying biological plausibility.

## Conclusion

In light of our research findings, we strongly recommend that health professionals and policymakers carefully consider a comprehensive set of interventions to alleviate the burden of pain among older adults. These interventions should be integrated into the National Programme for Health Care of the Elderly (NPHCE). It includes health education programs, promoting gender equity in health accessibility, initiating physical activity initiatives, providing nutritional support, and expanding palliative care services. It is crucial to customise these interventions to align with the distinct needs and preferences of the older adult population residing in regions and socioeconomic groups characterised by a high prevalence of pain. Effective implementation hinges on fostering close collaboration among public health professionals, healthcare providers, and local communities. This collaborative approach is essential to ensure that these initiatives are tailored to the specific context and well-received and impactful in improving the overall health and quality of life for older adults.

### Electronic supplementary material

Below is the link to the electronic supplementary material.

Supplementary Material 1

## Data Availability

The data is publicly available and can be accessed upon request at https://www.iipsindia.ac.in/content/LASI-data.

## Abbreviations

LASI: Longitudinal Ageing Study in India
BMI: Body Mass Index
MPCE: Monthly Per Capita Expenditure
SC: Scheduled Caste
ST: Schedule Tribe
OBC: Other Backward Caste
