# Supplementary material for: Socioeconomic variation in the prevalence of pain by anatomical sites among middle-aged and older adults in India: a cross-sectional study
Source: BMC Geriatr. 2024 Feb 27;24:198. doi: 10.1186/s12877-024-04780-1 (PMC10900751; doi:10.1186/s12877-024-04780-1)
Supplement: Supplementary file 1 — Supplementary Material 1 [file 12877_2024_4780_MOESM1_ESM.docx]

| Appendix 1: Model Robustness Assessment for Socio-Economic Predictors of Pain Occurrence in different Anatomical Sites (Age Group: 45-59) | | | | |
| --- | --- | --- | --- | --- |
| **Socio-Demographic Characteristics** | **Any Pain** | **Back Pain** | **Joint Pain** | **Ankle/foot Pain** |
|  | **AORs (95% CI)** | **AORs (95% CI)** | **AORs (95% CI)** | **AORs (95% CI)** |
| **Sex** |  |  |  |  |
| Male® |  |  |  |  |
| Female | 1.61*** (1.42 - 1.83) | 1.48*** (1.26 - 1.73) | 1.49*** (1.29 - 1.72) | 1.62*** (1.32 - 2.00) |
| **Residence** |  |  |  |  |
| Rural® |  |  |  |  |
| Urban | 0.98 (0.86 - 1.12) | 0.97 (0.81 - 1.15) | 0.99 (0.83 - 1.18) | 1.05 (0.89 - 1.24) |
| **Years of Education** |  |  |  |  |
| No schooling® |  |  |  |  |
| < 5 years | 0.95** (0.84 - 1.07) | 0.91 (0.77 - 1.07) | 0.95 (0.83 - 1.08) | 0.89 (0.75 - 1.06) |
| 5-9 years | 0.77*** (0.66 - 0.88) | 0.81** (0.65 - 0.99) | 0.71*** (0.62 - 0.82) | 0.81** (0.67 - 0.99) |
| ≥ 10 years | 0.68*** (0.60 - 0.78) | 0.70*** (0.57 - 0.86) | 0.68*** (0.56 - 0.82) | 0.67** (0.47 - 0.95) |
| **Currently Married** |  |  |  |  |
| No® |  |  |  |  |
| Yes | 1.09 (0.94 - 1.27) | 1.15** (1.00 - 1.32) | 0.98 (0.84 - 1.14) | 0.96 (0.78 - 1.18) |
| **Caste** |  |  |  |  |
| Scheduled caste® |  |  |  |  |
| Scheduled tribe | 1.23** (1.01 - 1.48) | 1.32** (1.09 - 1.59) | 1.16 (0.95 - 1.41) | 1.12 (0.84 - 1.49) |
| Other Backward Class | 1.09 (0.96 - 1.24) | 1.05 (0.94 - 1.18) | 1.16** (1.02 - 1.31) | 0.97** (0.81 - 1.16) |
| Others | 1.00 (0.87 - 1.16) | 0.98 (0.84 - 1.15) | 0.97 (0.83 - 1.13) | 1.00* (0.84 - 1.19) |
| **Religion** |  |  |  |  |
| Hindu® |  |  |  |  |
| Muslim | 1.00 (0.87 - 1.15) | 1.24** (1.07 - 1.44) | 0.90 (0.79 - 1.03) | 0.91 (0.68 - 1.20) |
| Christian | 0.86 (0.49 - 1.51) | 1.06 (0.72 - 1.54) | 0.91 (0.54 - 1.53) | 0.49** (0.30 - 0.81) |
| Others | 1.33** (1.08 - 1.62) | 1.17* (0.97 - 1.42) | 1.11 (0.88 - 1.38) | 1.23* (0.97 - 1.56) |
| **MPCE quintile** |  |  |  |  |
| Poorest® |  |  |  |  |
| Poorer | 1.12 (0.96 - 1.30) | 1.06 (0.92 - 1.23) | 1.10 (0.93 - 1.30) | 1.15 (0.94 - 1.41) |
| Middle | 1.09 (0.90 - 1.32) | 0.94 (0.81 - 1.09) | 1.08 (0.88 - 1.32) | 1.41** (1.06 - 1.87) |
| Richer | 1.11 (0.94 - 1.30) | 1.14 (0.96 - 1.34) | 1.03 (0.88 - 1.20) | 1.41** (1.15 - 1.72) |
| Richest | 1.42** (1.13 - 1.80) | 1.56***(1.19 - 2.05) | 1.39** (1.06 - 1.82) | 1.43** (1.09 - 1.89) |
| **Currently Working** |  |  |  |  |
| No® |  |  |  |  |
| Yes | 0.88** (0.80 - 0.97) | 0.98 (0.88 - 1.10) | 0.84*** (0.76 - 0.93) | 0.81** (0.69 - 0.94) |
| **BMI** |  |  |  |  |
| Underweight (≤ 18.5) ® |  |  |  |  |
| Normal (18.5 - 25.0) | 1.28 (1.11 - 1.47) | 1.11 (0.96 - 1.30) | 1.29*** (1.12 - 1.48) | 1.15* (1.00 - 1.32) |
| Overweight (25.0 - 30.0) | 1.69*** (1.42 - 2.00) | 1.15*** (0.97 - 1.35) | 1.79*** (1.51 - 2.12) | 1.66** (1.31 - 2.09) |
| Obese (>30) | 2.13*** (1.73 - 2.61) | 1.33** (1.03 - 1.71) | 2.07*** (1.66 - 2.58) | 1.91** (1.57 - 2.31) |
| **Smoking history** |  |  |  |  |
| No® |  |  |  |  |
| Yes | 1.22 (1.10 - 1.35) | 1.13* (1.00 - 1.29) | 1.13** (1.01 - 1.27) | 1.21** (1.05 - 1.39) |
| **Alcohol History** |  |  |  |  |
| No® |  |  |  |  |
| Yes | 1.07 (0.95 - 1.21) | 1.06 (0.93 - 1.21) | 1.05 (0.92 - 1.19) | 0.96 (0.80 - 1.16) |
| **Physically Active** |  |  |  |  |
| No® |  |  |  |  |
| Yes | 0.99 (0.90 - 1.10) | 0.95 (0.85 - 1.05) | 1.04 (0.93 - 1.17) | 0.86** (0.76 - 0.97) |
| note: MPCE is an abbreviation for monthly per capita consumption expenditure and BMI refers to body mass index. | | | | |
| *** significant at 0.001 level; ** at 0.05 level; * at 0.10 level | | | | |

| Appendix 2: Model Robustness Assessment for Socio-Economic Predictors of Pain Occurrence in different Anatomical Sites (Age Group: 60+) | | | | |
| --- | --- | --- | --- | --- |
| **Socio-Demographic Characteristics** | **Any Pain** | **Back Pain** | **Joint Pain** | **Ankle/foot Pain** |
|  | **AORs (95% CI)** | **AORs (95% CI)** | **AORs (95% CI)** | **AORs (95% CI)** |
| **Sex** |  |  |  |  |
| Male® |  |  |  |  |
| Female | 1.47*** (1.33 - 1.63) | 1.65*** (1.48 - 1.84) | 1.28*** (1.16 - 1.41) | 1.23** (1.07 - 1.42) |
| **Residence** |  |  |  |  |
| Rural® |  |  |  |  |
| Urban | 0.91 (0.81 - 1.03) | 1.01 (0.89 - 1.15) | 0.94 (0.84 - 1.06) | 1.14 (0.94 - 1.37) |
| **Years of Education** |  |  |  |  |
| No schooling® |  |  |  |  |
| < 5 years | 0.93 (0.82 - 1.05) | 0.97 (0.83 - 1.14) | 0.84** (0.74 - 0.95) | 0.87 (0.73 - 1.04) |
| 5-9 years | 0.73** (0.63 - 0.84) | 0.72*** (0.60 - 0.85) | 0.72** (0.61 - 0.84) | 0.79** (0.63 - 0.98) |
| ≥ 10 years | 0.54** (0.45 - 0.65) | 0.64** (0.48 - 0.85) | 0.51*** (0.43 - 0.60) | 0.64** (0.46 - 0.90) |
| **Currently Married** |  |  |  |  |
| No® |  |  |  |  |
| Yes | 1.06 (0.94 - 1.20) | 1.13** (1.01 - 1.26) | 1.05 (0.93 - 1.19) | 0.96 (0.78 - 1.17) |
| **Caste** |  |  |  |  |
| Scheduled caste® |  |  |  |  |
| Scheduled tribe | 1.14 (0.93 - 1.40) | 1.12 (0.92 - 1.38) | 1.14 (0.92 - 1.41) | 1.06 (0.82 - 1.36) |
| Other Backward Class | 1.09 (0.96 - 1.25) | 1.01 (0.88 - 1.16) | 1.02 (0.90 - 1.16) | 0.94 (0.78 - 1.13) |
| Others | 1.10 (0.94 - 1.28) | 1.02 (0.85 - 1.21) | 0.95 (0.81 - 1.11) | 1.08 (0.86 - 1.35) |
| **Religion** |  |  |  |  |
| Hindu® |  |  |  |  |
| Muslim | 0.84** (0.69 - 1.01) | 0.93 (0.76 - 1.13) | 0.86** (0.74 - 1.00) | 0.74** (0.61 - 0.90) |
| Christian | 1.15 (0.88 - 1.50) | 1.15 (0.87 - 1.50) | 1.06 (0.85 - 1.31) | 0.57** (0.40 - 0.82) |
| Others | 0.88 (0.70 - 1.10) | 0.94 (0.78 - 1.13) | 0.88 (0.70 - 1.12) | 0.80* (0.62 - 1.03) |
| **MPCE quintile** |  |  |  |  |
| Poorest® |  |  |  |  |
| Poorer | 1.13** (0.99 - 1.30) | 1.10 (0.97 - 1.25) | 1.19** (1.05 - 1.35) | 1.21** (1.02 - 1.44) |
| Middle | 1.05 (0.92 - 1.21) | 1.05 (0.90 - 1.23) | 1.02 (0.90 - 1.17) | 1.18** (1.00 - 1.40) |
| Richer | 1.10 (0.92 - 1.32) | 1.07 (0.88 - 1.31) | 1.08 (0.92 - 1.26) | 1.34** (1.07 - 1.68) |
| Richest | 1.23** (1.04 - 1.45) | 1.29** (1.08 - 1.55) | 1.30** (1.11 - 1.54) | 1.33** (1.07 - 1.65) |
| **Currently Working** |  |  |  |  |
| No® |  |  |  |  |
| Yes | 0.84*** (0.76 - 0.92) | 0.97*** (0.87 - 1.08) | 0.73*** (0.66 - 0.81) | 0.80*** (0.71 - 0.90) |
| **BMI** |  |  |  |  |
| Underweight (≤ 18.5) ® |  |  |  |  |
| Normal (18.5 - 25.0) | 1.21*** (1.09 - 1.34) | 1.03 (0.94 - 1.14) | 1.23*** (1.12 - 1.36) | 1.11 (0.98 - 1.26) |
| Overweight (25.0 - 30.0) | 1.90*** (1.66 - 2.18) | 1.07 (0.93 - 1.24) | 1.85*** (1.62 - 2.10) | 1.64*** (1.38 - 1.95) |
| Obese (>30) | 2.82*** (2.10 - 3.77) | 1.60** (1.07 - 2.39) | 2.86*** (2.12 - 3.86) | 2.65*** (1.88 - 3.74) |
| **Smoking history** |  |  |  |  |
| No® |  |  |  |  |
| Yes | 1.20*** (1.09 - 1.32) | 1.20*** (1.09 - 1.33) | 1.12** (1.02 - 1.24) | 1.04 (0.92 - 1.18) |
| **Alcohol History** |  |  |  |  |
| No® |  |  |  |  |
| Yes | 1.07 (0.94 - 1.21) | 0.98 (0.85 - 1.14) | 1.09 (0.97 - 1.22) | 0.96 (0.80 - 1.14) |
| **Physically Active** |  |  |  |  |
| No® |  |  |  |  |
| Yes | 0.91** (0.82 - 1.01) | 0.92* (0.84 - 1.00) | 0.93 (0.84 - 1.03) | 0.95 (0.81 - 1.11) |
| note: MPCE is an abbreviation for monthly per capita consumption expenditure and BMI refers to body mass index. | | | | |
| *** significant at 0.001 level; ** at 0.05 level; * at 0.10 level | | | | |

| Appendix 3: State-wise estimates of pain prevalence by distinct anatomical sites. | | | | |
| --- | --- | --- | --- | --- |
| **States** | **Any Pain % (95% CI)** | Back Pain  % (95% CI) | **Joint Pain % (95% CI)** | **Ankle/foot Pain % (95% CI)** |
| **India** | **59.39 (58.35 - 60.43)** | **31.68 (30.68 - 32.69)** | **47.18 (45.95 - 48.40)** | **19.87 (18.77 - 20.98)** |
| **Jammu & Kashmir** | 66.91 (62.53 - 71.28) | 44.56 (40.52 - 48.60) | 56.29 (51.01 - 61.56) | 14.87 (10.98 - 18.77) |
| **Himachal Pradesh** | 56.01 (51.64 - 60.37) | 30.78 (27.29 - 34.28) | 45.96 (41.39 - 50.54) | 12.15 (8.28 - 16.03) |
| **Punjab** | 65.61 (62.96 - 68.26) | 34.81 (31.69 - 37.93) | 53.70 (50.41 - 56.98) | 22.72 (20.15 - 25.30) |
| **Chandigarh** | 58.73 (55.78 - 61.68) | 32.61 (29.97 - 35.25) | 43.89 (40.20 - 47.57) | 20.49 (17.05 - 23.94) |
| **Uttarakhand** | 75.43 (72.31 - 78.56) | 46.93 (42.71 - 51.16) | 67.53 (64.33 - 70.74) | 20.17 (14.99 - 25.35) |
| **Haryana** | 67.37 (62.97 - 71.77) | 30.83 (26.46 - 35.20) | 54.51 (48.84 - 60.17) | 26.03 (22.82 - 29.23) |
| **Delhi** | 67.20 (61.50 - 72.90) | 27.25 (21.81 - 32.69) | 54.85 (47.98 - 61.72) | 21.02 (17.12 - 24.93) |
| **Rajasthan** | 53.02 (48.98 - 57.06) | 25.27 (22.40 - 28.13) | 42.34 (38.28 - 46.41) | 23.86 (20.01 - 27.71) |
| **Uttar Pradesh** | 58.26 (55.75 - 60.77) | 28.51 (26.60 - 30.43) | 48.35 (45.84 - 50.86) | 27.05 (24.35 - 29.76) |
| **Bihar** | 63.95 (60.80 - 67.10) | 39.29 (35.99 - 42.59) | 47.75 (43.81 - 51.70) | 17.95 (15.08 - 20.82) |
| **Sikkim** | 54.12 (48.47 - 59.76) | 45.57 (39.46 - 51.69) | 33.11 (28.15 - 38.07) | 06.46 (3.71 - 9.20) |
| **Arunachal Pradesh** | 70.26 (64.61 - 75.92) | 60.82 (54.62 - 67.02) | 43.62 (35.36 - 51.89) | 03.52 (1.68 - 5.35) |
| **Nagaland** | 77.90 (71.87 - 83.94) | 66.21 (59.13 - 73.29) | 60.19 (53.34 - 67.03) | 02.51 (1.21 - 3.80) |
| **Manipur** | 79.12 (74.36 - 83.87) | 71.16 (66.00 - 76.32) | 45.32 (40.57 - 50.07) | 13.23 (8.45 - 18.00) |
| **Mizoram** | 67.53 (58.66 - 76.39) | 56.06 (46.09 - 66.03) | 34.03 (30.92 - 37.13) | 08.76 (5.26 - 12.26) |
| **Tripura** | 57.83 (53.14 - 62.52) | 42.03 (37.41 - 46.65) | 33.28 (28.17 - 38.40) | 09.99 (7.69 - 12.29) |
| **Meghalaya** | 55.70 (46.20 - 65.20) | 46.50 (36.88 - 56.11) | 37.02 (30.03 - 44.02) | 05.37 (3.42 - 7.33) |
| **Assam** | 64.52 (60.79 - 68.26) | 39.88 (36.05 - 43.72) | 54.00 (50.25 - 57.74) | 08.94 (7.15 - 10.74) |
| **West Bengal** | 43.22 (38.76 - 47.68) | 20.94 (17.80 - 24.07) | 26.78 (22.66 - 30.90) | 11.12 (8.99 - 13.25) |
| **Jharkhand** | 68.28 (65.30 - 71.27) | 37.99 (35.10 - 40.87) | 54.63 (51.10 - 58.17) | 25.46 (21.54 - 29.38) |
| **Odisha** | 63.48 (61.11 - 65.85) | 25.45 (23.04 - 27.85) | 54.98 (52.33 - 57.63) | 14.65 (12.39 - 16.91) |
| **Chhattisgarh** | 52.84 (48.21 - 57.47) | 29.28 (25.81 - 32.76) | 42.75 (37.69 - 47.81) | 13.78 (11.22 - 16.33) |
| **Madhya Pradesh** | 66.30 (61.87 - 70.73) | 36.01 (31.25 - 40.76) | 59.91 (55.73 - 64.08) | 32.14 (27.73 - 36.56) |
| **Gujarat** | 45.07 (41.91 - 48.23) | 18.32 (16.24 - 20.40) | 35.02 (31.10 - 38.94) | 15.40 (13.09 - 17.71) |
| **Daman and Diu** | 45.59 (38.04 - 53.15) | 20.84 (15.42 - 26.26) | 34.84 (26.44 - 43.23) | 18.61 (15.59 - 21.63) |
| **Dadra and Nagar Haveli** | 52.41 (46.39 - 58.43) | 30.37 (25.53 - 35.22) | 41.95 (35.98 - 47.92) | 16.22 (12.88 - 19.55) |
| **Maharashtra** | 66.11 (63.43 - 68.78) | 41.81 (39.12 - 44.50) | 42.72 (39.67 - 45.76) | 26.93 (24.76 - 29.09) |
| **Andhra Pradesh** | 67.21 (63.79 - 70.62) | 31.46 (27.62 - 35.29) | 58.76 (54.98 - 62.54) | 14.43 (11.57 - 17.30) |
| **Karnataka** | 68.36 (64.22 - 72.49) | 39.36 (32.28 - 46.45) | 59.90 (54.63 - 65.16) | 25.61 (17.82 - 33.40) |
| **Goa** | 61.02 (57.07 - 64.98) | 34.30 (30.67 - 37.94) | 37.05 (33.06 - 41.04) | 20.71 (17.37 - 24.06) |
| **Lakshadweep** | 55.43 (45.82 - 65.04) | 38.67 (30.91 - 46.43) | 40.54 (33.35 - 47.72) | 08.16 (5.64 - 10.67) |
| **Kerala** | 62.48 (57.51 - 67.46) | 40.70 (35.70 - 45.70) | 49.10 (45.34 - 52.86) | 21.06 (17.65 - 24.46) |
| **Tamil Nadu** | 41.30 (37.33 - 45.27) | 14.49 (11.77 - 17.22) | 35.73 (32.05 - 39.40) | 03.58 (2.32 - 4.84) |
| **Puducherry** | 53.55 (50.35 - 56.76) | 24.01 (20.35 - 27.67) | 47.18 (43.01 - 51.36) | 06.65 (4.73 - 8.57) |
| **Andaman and Nicobar** | 68.37 (63.46 - 73.28) | 42.14 (36.50 - 47.79) | 48.82 (43.43 - 54.21) | 19.21 (15.05 - 23.38) |
| **Telangana** | 69.55 (66.21 - 72.89) | 40.74 (36.39 - 45.09) | 59.46 (55.64 - 63.27) | 16.45 (13.15 - 19.76) |

**Supplementary Text 1: Age-sex Adjustment Method**

The following text was adapted from that appearing as Supplementary Text S4 in Mohanty et al. (2021) (Mohanty et al., 2021)

We adjusted pain prevalence estimates for age and sex by using the age-sex composition of the nationally representative total sample as the reference. For example, to obtain age-sex-adjusted prevalence by state (figure 3 & 4), we estimated a logistic regression of pain as an outcome on a complete set of state indicators (fixed effects) and 36 sex-specific age group (<45 years, 45-46, 46-47, 48-49, …, 69-70, 71-75, 76-80, 81-85, and 85+) indicators (fixed effects) with sample weights applied. Then, for each state, we averaged the predicted prevalence of pain if located in that state's overall sample participants, i.e. the average adjusted prevalence. This gave an estimate of the prevalence in a state if its age-sex composition was the same as that of the whole sample, which was representative of the national population aged 45+ when sample weights were applied.

References

Mohanty, S. K., Pedgaonkar, S. P., Upadhyay, A. K., Kämpfen, F., Shekhar, P., Mishra, R. S., . . . O’Donnell, O. (2021). Awareness, treatment, and control of hypertension in adults aged 45 years and over and their spouses in India: A nationally representative cross-sectional study. *PLOS Medicine*, *18*(8), e1003740. <https://doi.org/10.1371/journal.pmed.1003740>
